# Polymer mimics of biomacromolecular antifreezes

**DOI:** 10.1038/s41467-017-01421-7

**Published:** 2017-11-16

**Authors:** Caroline I. Biggs, Trisha L. Bailey, Ben Graham, Christopher Stubbs, Alice Fayter, Matthew I. Gibson

**Affiliations:** 1 Department of Chemistry, University of Warwick, Coventry, CV4 7AL UK; 2 Warwick Medical School, University of Warwick, Coventry, CV4 7AL UK

## Abstract

Antifreeze proteins from polar fish species are remarkable biomacromolecules which prevent the growth of ice crystals. Ice crystal growth is a major problem in cell/tissue cryopreservation for transplantation, transfusion and basic biomedical research, as well as technological applications such as icing of aircraft wings. This review will introduce the rapidly emerging field of synthetic macromolecular (polymer) mimics of antifreeze proteins. Particular focus is placed on designing polymers which have no structural similarities to antifreeze proteins but reproduce the same macroscopic properties, potentially by different molecular-level mechanisms. The application of these polymers to the cryopreservation of donor cells is also introduced.

## Abstract (web summary)

Ice crystal growth is a major problem in cell and tissue cryopreservation for transplantation, transfusion, icing of aircraft wings and many other applications. Here the authors review the emerging field of synthetic macromolecular mimics of antifreeze proteins that can be used overcome such problems.

## Introduction

Water is fundamental to all life on our planet, and despite it having a freezing point of 0 ˚C, Nature has evolved a series of unique adaptations to enable life to flourish in sub-zero climates, at high altitudes and at the Earth’s poles. Such extremophiles include the wood frog (*Lithobates sylvaticus*) which can freeze solid over winter, tardigrades which can be desiccated and rehydrated and cold tolerant plants^1, 2^. The mechanisms of these cryoprotectants are varied, from enabling freeze-tolerance (being able to be frozen and then thawed) to freeze avoidance (preventing ice forming) and even freeze promotion (as a predatory mechanism)^3–6^.

**Fig. 1:**
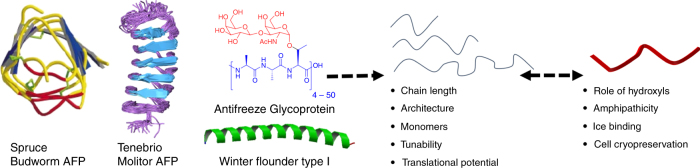
General concept of this review. Simplification of antifreeze proteins and antifreeze glycoproteins to fully synthetic polymers. This figure is adapted from Gibson (2010)^24^ with permission of The Royal Society of Chemistry. This image is not included under the creative commons licence for this article

One particular adaptation is the production of macromolecular antifreezes (proteins and polysaccharides) which modulate ice formation and growth, and are found in freeze avoidant organisms. These can be broadly split into the antifreeze proteins (AFPs) and antifreeze glycoproteins (AFGPs). AFGPs are highly conserved, with a relatively simple repeat tripeptide structure and a disaccharide on every third amino acid, but are produced in a range of chain lengths. Conversely, AFPs are far more diverse, with several subdivisions, and can assume different structures; from beta barrels to alpha helices and vary in size (Fig. 1). They all have a few core properties (discussed in detail below) including the ability to inhibit ice recrystallization, shape ice crystals into unusual morphologies and to depress the freezing point in a non-colligative manner. The relative magnitude of each effect varies between individual AF(G)Ps and the exact mechanisms, involving ice-face recognition, are still under investigation. What is clear, is that the ability to tune and modify ice growth and formation has the potential for huge industrial and societal impact. For example, ice adhesion limits the performance of wind farms by up to 50%^7^, is a major problem for aircraft^8^ and even impacts our understanding of how biological components affect our climate^9, 10^.

Some AFPs have already found application in improving the texture of ice cream products by preventing ice crystal growth, and other food uses are under investigation^4, 11^. A key potential field where AF(G)Ps could be applied is in cell and tissue cryopreservation where ice recrystallization is a major problem^12–14^. The availability of high quality cells and tissues underpins all modern biomedical science and drug screening, and is crucial for emerging regenerative medicine applications. Progenitor (stem) cells remain challenging to cryopreserve, requiring large amounts of organic solvents which give less than 100% cell recovery and can affect their future differentiation pathways^15^. AF(G)Ps are not always suitable for cryopreservation due to potential immunogenicity and toxicity concerns^16^, expense of production and the formation of needle like ice crystals (dynamic ice shaping), which is undesirable for cell cryopreservation, leading to mixed successes and failures^12, 13, 17^. There is a clear rationale to develop synthetic mimics with tunable and tailored function.

In the past 20 years, huge advances have been made in synthetic polymer chemistry, which now enables the design and precise synthesis of a plethora of complex architectures incorporating nearly any functional group^18^ and even some progress towards introducing monomer sequence has been made^19^. This has enabled the emergence of synthetic materials which have protein-like function but with the scalability and tunability associated with polymers. For example, nanoscale drug delivery devices from block copolymers^20^, biochemically responsive polymers^21^, glycopolymer mimics of natural glycoproteins and polysaccharides^22^ and nucleobase containing polymers^23^.

The aim of this perspective is to introduce the rapidly emerging field of macromolecular cryoprotectants^24^—polymers designed to mimic the complex function of antifreeze (glyco)proteins, without necessarily mimicking their chemical structure. This focus on function, not structure, is enabling the identification of new materials with unexpected properties. These synthetic entities are providing new insights into the mechanisms of action, enabling translation towards real world applications and a particular focus will be placed on their application in cellular cryopreservation. The physical properties of AF(G)Ps and how they are measured will be summarized, along with small molecule mimetics, followed by a comprehensive summary of the state of the art within macromolecular mimics.

## Macroscopic Properties of Antifreeze(glyco) Proteins

**Fig. 2:**
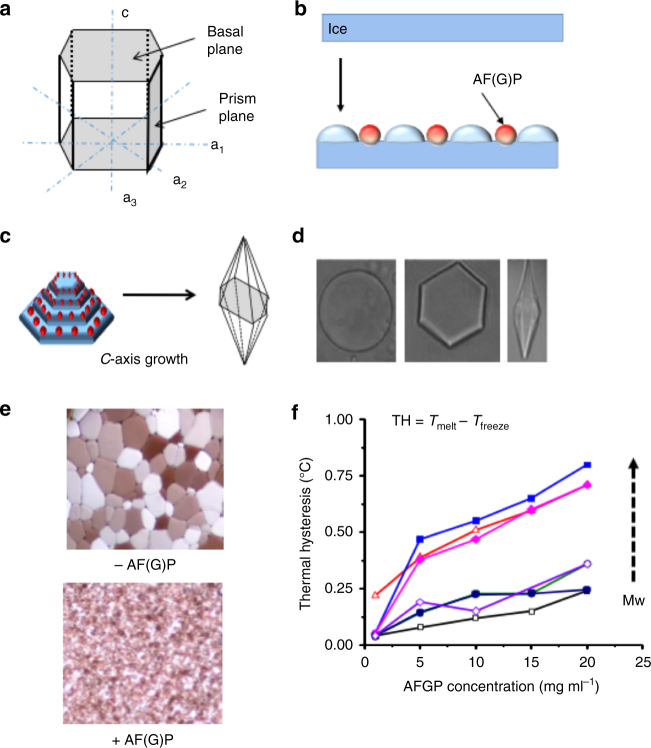
Interaction of antifreeze proteins with ice. **a** Hexagonal ice crystal; **b** Adsorption-inhibition where AF(G)Ps bind to ice, causing local curvature and hence growth inhibition; **c** Preferential *c*-axis (basal) growth when prism planes inhibited; **d** Different morphologies observed for single ice crystals; **e** Micrographs of ice crystals after annealing with/without antifreeze proteins showing IRI activity; **f** TH activity for different molecular weight AFGPs (2.6–24 kDa)^4, 33^. **a**–**d** are reproduced from Gibson (2010)^24^ with permission of The Royal Society of Chemistry. These images are not included under the creative commons licence for this article. **f** was created using the raw data from Wu et al. (2001)^33^

The addition of AF(G)Ps to aqueous solutions significantly affects both the ice formation and growth processes, with 3 key macroscopic effects associated with growth (note; nucleation is a separate property)^10^; dynamic ice shaping (DIS); the modification of the morphology of a growing ice crystal; ice recrystallization inhibition (IRI); crystal growth (Ostwald ripening) is kinetically suppressed such that growth is inhibited; thermal hysteresis (TH)**;** a non-equilibrium depression of the freezing point, resulting in a lower freezing than melting point, which is greater than what is expected on a colligative basis (Fig. 2f)^24–26^. While the details of the assays and ice–protein interactions are beyond the scope of this review it is necessary to include core techniques to aid understanding.

Early work in this field focused on AF(G)P function in relation to TH and DIS activity, which remain the most studied macroscopic properties. TH and DIS appear to be interlinked with all proteins which show TH also demonstrating DIS, although the exact magnitude of each property varies between proteins. This is most often explained by an adsorption-inhibition mechanism (Fig. 2a, b)^26, 27^. Without AF(G)Ps the curvature of the crystal is unaffected and a round flat crystal is produced (Fig. 2b). When AF(G)Ps are adsorbed onto a particular plane of ice, the local surface curvature increases, and hence an increase in vapour pressure, resulting in a lowering of melting point and water molecules being unable to assimilate into surface pockets in the ice^28^. By binding to the fastest growing plane (prism) the observed growth rate is slowed and progresses from the basal plane and hence shaping occurs, giving rise to the unique morphologies (Fig. 2c, d)^29^. Early work in the field, using hemispherical etching and ice plane affinity, supported these hypotheses, although they do not unravel the actual ice–protein interactions^30^. It is important to highlight that structural biology tools, probing protein structure and function, including single crystal structure determination and SAXS are providing insights into the structure of AFPs and have revealed the ice-binding domains in many cases, along with anchored clathrates, which appear to mediate the interaction with the ice. These details are beyond the scope of this review, but readers are pointed to several articles on the subject^31, 32^.

While thermal hysteresis and dynamic ice shaping appear correlated, the third property (IRI) appears to operate independently, with little or no correlation to the magnitude of TH activity^11^. For example, AFPs such as TmAFP, winter flounder type I-Hyp and *M*. *primoryenisis* are considered hyperactive, with high TH activity, but show relatively low IRI activity. Conversely AFGPs and winter flounder type I are moderately TH active but with high IRI^34^. These observations indicate that multiple molecular-level mechanisms may be present, and exploited differentially by the different proteins. The gold standard method to test for TH and DIS activity is nanoliter osmometry^25, 35^. Samples are frozen and then slowly melted until a single crystal remains. To determine the TH of a sample the crystal is cooled further until there is a sudden burst in growth. TH is defined as the difference between the melting point and non-equilibrium freezing point^4, 33^. There are limitations in determining TH in this way, mainly the technical challenges of avoiding burst growth and precise temperature control required, alongside the relatively high concentrations of material (>20 mg mL^−1^) needed to induce this effect. Sonocrystallization is a method proposed by Voets et al. to overcome these issues and allow the measurement of freezing and melting points in one experiment. The method involves supercooling a solution before the application of a short ultrasound pulse, which induces nucleation and freezing. The sample is then melted for melting point determination. Data obtained from sonocrystallization links to TH though does not scale with values obtained from nanoliter osmometry, which may provide new insights or indicate it is probing different molecular-level processes^25^. Low temperature solid-state NMR is emerging as a new tool in the field as it is possible to monitor the liquid water phase, the ice and the protein component all in the same experimental set-up.^1^H-^1^H cross-saturation and cross studies of frozen AFP:ice solutions have recently provided detailed structural information regarding the AFP:ice interface. These studies present solid-state NMR as a technique which is likely to lead to breakthroughs in the near future^36^.

The ‘splat cooling’ IRI assay is widely used to probe recrystallization^37^. In short, this method involves the formation of a polynucleated ice wafer by dropping a small volume of a buffered solution containing the AFP/inhibitor onto a pre-cooled surface. The ice crystals are then annealed at a sub-zero temperature, above the eutectic phase transition, and the growth of the ice crystals monitored. Due to the large number (100’s) of ice crystals obtained, various image analysis methods have been developed to facilitate this, but due to the non-regular size and shape of the ice crystals this remains non-trivial^38^. The length of time of the annealing will also affect the outcome, as this is only a kinetic slowing with slow growth still occurring.

A related assay using high concentrations of sucrose (40 wt%) is also used to study growth in complex media with the advantage of simpler image analysis due to a lower density of ice crystals being obtained and does not require apparatus to ‘splat’ the droplet^39^. Microcapillary methods for screening for IRI have been shown by Davies and co-workers, with the advantage of allowing the sample to be archived^40^. It is very important to note, that when interpreting IRI data, either crystal area (mean grain size) or crystal length (MLGS) are used as measures. As area has a squared term (e.g. nm^2^) these values tend to be smaller at equal activity. It is important to consider this when comparing inhibitors. As a guide, 40% MLGS would be equivalent to 16% MGS.

The following sections will discuss the synthesis and application of various AF(G)P mimetic strategies, focusing on the macromolecular mimics, and their application to cellular cryopreservation, while referring to their activity in the above-named assays.

## Small Molecule and Peptide Based Mimics

AFPs are routinely produced by recombinant expression methods or, for shorter sequences, by solid phase synthesis^6, 39^. AFGPs, which show greater IRI activity than AFPs, are harder to access as bacterial protein expression does not routinely enable the installation of the post-translation modification (in this case the disaccharide) necessitating chemical synthesis^41^. Therefore, efforts have been made to use chemical synthesis as a route to accessing these materials.

The total chemical synthesis of AFGPs has mainly focused on AFGP-8, the shortest naturally occurring fraction (Mw = 2700 g mol^−1^), Table 1 ^42^. Nishimura and co-workers reported the most comprehensive study of AFGPs to date^43^. In this work 10 different carbohydrates and different chain lengths were synthesized via a polymerization/fractionation approach. Interestingly, only TH and DIS were tested as an indicator of activity. It was concluded that an *N*-acetyl sugar, the γ-methyl on threonine and an α-glycosidic linkage were all essential components for TH and DIS to be retained. Solution phase NMR analysis revealed a largely unstructured secondary structure similar to that of a polyproline II helix (as seen in native AFGP), but interestingly all the glycans were presented on a single face creating an amphipathic structure. This is remarkably similar to crystal structures for AFPs which have clearly segregated hydrophobic (ice binding) and hydrophilic domains, suggesting that certain generic macro-scale features are crucial for function, rather than just the primary sequence; this is a key observation for the design of polymer mimics, later in this review. While the above observations were conclusive, in a sense, the study omitted any IRI activity testing (Table 1). This omission meant the role of structural simplifications and the minimum requirements for IRI could not be drawn, leading to conclusions that simplified AFGPs could not be designed, and hence synthetic mimics might not be so accessible—which as this review will highlight, was an incorrect assumption.

**Table 1:** Peptide/small molecule mimics with distinct IRI and TH behaviour

	

*MGS* mean grain size determined as the average area of each ice crystal, *MLGS* mean largest grain size, which reflects the length of the largest ice crystals

Ben and co-workers reported that glycopeptides bearing a *C*-linked galactosyl-serine and glycine (i.e. non stereogenic) backbone, rather than alanine, were highly potent IRI’s, functioning below 0.05 µM, which is more active than AFP Type III^16, 45^. Interestingly, no thermal hysteresis or dynamic ice shaping activity was observed, providing the first evidence that these properties were resolvable from IRI, which is uniquely tolerant to structural deviations from the core AFGP motif. Further studies from Ben and co-workers have explored the tolerance of glycans and backbone hydrophilicity effects on activity in detail, with galactose being preferred in general, and hydrophilic modifications to the backbone reducing activity (Table 1)^16, 47^. Inspired by the polyproline II helix of AFGP, glycosylated poly(proline) was also synthesized as a peptide mimetic and tested for activity; surprisingly it displayed greater activity without the glycan (as hydroxyproline) than with, highlighting the challenge associated with rational IRI synthesis, and indicating the ambiguity of the role of the sugar in AFGP; functional, or structural (to direct folding)^48^.

**Fig. 3:**
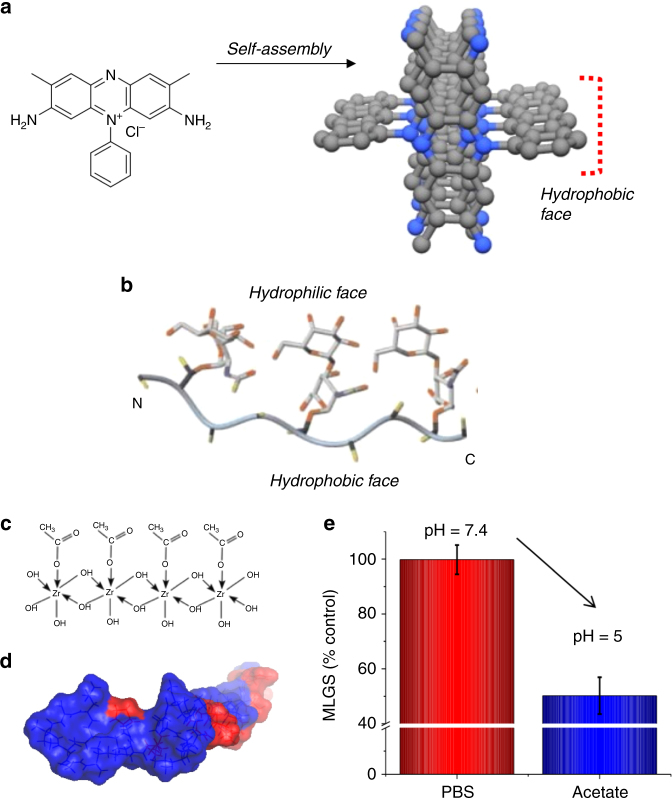
Amphipathic domains in IRI-active compounds. **a** Safranin O^53^; **b** Solution NMR structure of AFGP-8^59^; **c** Postulated structure of ZrOAc^56^; **d** Nisin A simulation with hydrophilic (red) and hydrophobic (blue) domains indicated^58^; **e** Modulation of Nisin A IRI by solution pH. **a** is adapted with permission from Drori et al. (2016)^53^. Copyright (2016) American Chemical Society. This image is not included under the creative commons licence for this article. **b** is reproduced from Tachibana et al. (2004)^43^. Copyright (2004) WILEY-VCH Verlag GmbH & Co.KGaA, Weinheim. This image is not included under the creative commons licence for this article. **c** is reproduced from Mizrahy et al. (2013)^56^ published under CC-BY4.0 at PLOS. **d**, **e** are reproduced from Mitchell et al., (2015)^58^ published under CC-BY4.0 at ACS

Carbohydrates alone have some weak IRI, with the hypothesis that their degree of hydration correlates with activity, but whether this relationship holds when attached to the AFGP backbone remains unknown^44, 49^. Ben and co-workers were the first to report the synthesis of low molecular weight carbohydrate derived small molecules possessing IRI activity^46, 49, 50^. Inspired by the obvious presentation of hydrophobic faces in AF(G)Ps, (Fig. 3b) installation of hydrophobic groups onto the anomeric position was a route to increase activity. Alkyl (octyl/nonyl/decyl) galactosides were found to inhibit recrystallization at 5.5 mM, compared to the free sugar which failed to inhibit fully even at 22 mM. A key design criteria emerged, with longer alkyl substituents being more active, until micellization occurs, at which point the hydrophobic domains are no longer presented, as they are internalized within the core^49, 51^.

It is clear from these non-peptide studies that the sugar could be seen as acting as a hydrophilic moiety, complementary to the hydrophobic unit necessary to induce activity, and is not in itself playing an essential recognition derived role. Taking this concept further, lysine based surfactants with non-sugar components and a charged headgroup were engineered such that they have IRI activity, indicating that a sugar group is a useful but not essential motif^52^.

The essential requirement for IRI to have amphipathic structures is a challenging design criteria, as hydrophobicity tends to drive aggregation or self-assembly which results in only hydrophilic surfaces being presented. Drori and co-workers screened a range of sparingly soluble organic dyes, which have flat planar structures ideal for self-assembly for TH/DIS/IRI activity. Safranin O, was identified as being a highly potent IRI-active species, inhibiting all ice growth at 4.2 mM (1.47 mg mL^−1^), which is a remarkable level of activity for a small molecule. Detailed studies revealed it was actually forming long facially amphipathic fibres similar to a rigid AFP (Fig. 3a, b)^53^. Amphipathic enantiomerically pure metallohelicies have also been found to be IRI active^54^. An unexpected IRI controlling compound is zirconium acetate (ZrAc) (Fig. 3d). ZrAc was observed by Deville and co-workers, to give strange pore shapes during ice-templated materials synthesis. Further experiments showed it to be a potent ice shaper and a very potent IRI (150 mM, 13.3 mg mL^−1^)^55^. ZrAc is a well-known co-ordination polymer, and it is hypothesized to have a solution structure^56^ related to poly(vinyl alcohol) (PVA), a highly potent IRI (see later) which may explain activity^57^. Nisin A, an antimicrobial peptide with pH switchable amphiphilicity was used to further exemplify the role of segregating domains, and the first example of a ‘stimuli-responsive’ IRI (Fig. 3d, e)^58^. The above clearly shows that a rather diverse set of materials have been shown to have activity, but clear structure-property relationships are still missing, except for the key observation that higher molecular weights seem to lead to more activity which provides inspiration for the use of synthetic polymers.

## Synthetic Polymer Materials with Antifreeze Protein-Like Function

The previous sections have served to highlight the attempts to reproduce the function of natural macromolecular antifreezes, especially in inhibiting ice recrystallization, and small molecule mimetics. There are clearly still no design rules and the de novo design of new non-peptide IRI-active compounds remains elusive. Compared to small molecules, synthetic polymers are often low cost, and can be assembled from a vast range of monomers and into diverse topologies and architectures offering vast scope for modulating function.

However, given the precise folding of AF(G)Ps and the largely unstructured nature of synthetic polymers this would seem to be an ambitious goal. In 1995, however, Knight observed that PVA, was a potent IRI but did not display significant thermal hysteresis^37^. This was one of the first instances of a synthetic polymer displaying such a property, and can be considered the birth of this field. Inada et al. evaluated the AF(G)P-like properties using commercial PVA samples^60^. They found that PVA had a molecular weight dependence on IRI activity and that on a molar basis had activity comparable to the shortest AFGPs. A key point to note here is that due to the high molar mass (Mw) of the polymers they were actually 10–100 fold less active than AFGP on a mass basis, but still remain the most active synthetic polymers reported today. Due to the activity of PVA, a simple assumption would be that any poly(hydroxylated) polymer could show IRI, as AFGPs are also poly-ols to a first approximation. Gibson et al. have tested various polysaccharides and glycopolymers which were found to be weak IRIs; in short, a poly-ol alone is not the minimum required feature of a synthetic IRI^61, 62^. Koop et al. have postulated that the spacing between hydroxyls on PVA are a good match for the basal plane of a growing ice crystal and hence may explain its activity^63^. However, this does not take into account the observations with small-molecule IRIs about the role of amphiphilicity, and that an AFP’s ice-binding face is actually the most hydrophobic, with the hydrophilic face directed into the unfrozen water layer; in short, this is still not clear, but multiple molecular-level mechanisms could give rise to the same macroscopic affects and it therefore warrants more study.

**Fig. 4:**
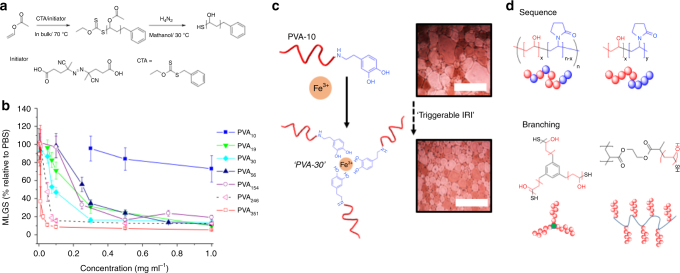
Well-defined PVA and IRI activity. **a** Synthesis of PVA by RAFT polymerization and deprotection with hydrazine^57^; **b** IRI activity of PVA as a function of degree of polymerization; **c** ‘Triggerable IRIs’ using catechol-terminated PVA and Fe^3+^ as the trigger; **d** Random verses block copolymers of PVA and poly(vinyl pyrrolidone) with only the latter showing IRI and star and comb-shaping PVA. **a** and **b** are adapted with permission from Congdon et al. (2013)^57^. Copyright (2013) American Chemical Society. These images are not included under the creative commons licence for this article. **c** was adapted from Phillips et al. (2016)^67^ with permission of The Royal Society of Chemistry. This image is not included under the creative commons licence for this article

The above studies were all conducted using commercial PVA samples; these are characterized by broad molecular weight distributions (Mw/Mn>2), low molecular weight contaminants (unless dialyzed before use) and the presence of 0–20 mol% acetate groups remaining from its synthesis from poly(vinyl acetate). This inhomogeneity meant that drawing structure-activity relationships was challenging, if not impossible. To enable a more systematic approach, Congdon et al. employed controlled radical polymerization (RAFT) to obtain well-defined PVA of predictable chain length and narrow molecular weight dispersity (Fig. 4a, b)^57^. By using hydrazine (rather than NaOH) to remove the acetate groups it was possible to obtain homogenous PVA. Selective re-acetylation, or co-polymerization with isopropenylacetate, revealed that incorporation of more than 20 mol% of any additional functionality lead to substantial reduction in the IRI activity. Crucially, this explains the inconsistent results from commercial PVAs due to their residual acetates, but also shows how sensitive the PVA structure is to structural modifications. Interestingly, similar effects are demonstrated by AFGP’s which lose activity if >35% of the hydroxyl groups are removed, but whether this is due to the loss of hydroxyl ‘function’ or a change in the folding of the protein has not been resolved^64^. Conversely, block co-polymerization of PVA appeared to be tolerated, with no change in activity. Fusion proteins of AFPs with *N*-terminus maltose binding protein have been found to have identical activity to free proteins also supporting that large chain-end modifications are tolerated^65^.

Inada’s original work had revealed a strong molecular weight dependence on activity^60^. Gibson and co-workers confirmed that even at relatively low molecular weights (<10,000 g mol^−1^) there was significant IRI. An increase in chain length generally increases IRI activity but the most startling increase was seen to be between DP 10 (~450 g mol^−1^) and DP 20 (~900 g mol^−1^) (Fig. 4b) where the PVA essentially switched from being inactive to active. This was the first demonstration of the critical point in terms of size being required to activate IRI activity and shows that the crucial length for activity is rather short, compared to e.g. AF(G)Ps and may give future insight into its mechanism of action^57^. This molecular weight dependence was also exploited to generate the first example of an ‘externally triggerable’ IRI. Catechol end-functional PVA was synthesized and upon addition of Fe^3+^ enabled formation of a supramolecular polymer with 3× molecular weight, and hence dramatically higher IRI (Fig. 4c)^66^. Such systems where an external stimulus can control IRI have not be widely explored, but a small molecule photo-reactive IRI has been shown, offering hope for spatiotemporal control over IRI.

A key advantage of using controlled RAFT is the ability to modulate polymer architecture and topology^18^. 3-armed, star branched PVA was found to have essentially identical activity to a 2-armed equivalent suggesting the third arm is redundant, and that total hydrodynamic volume rather than valency (number of hydroxyls) is a crucial parameter. Voets and co-workers synthesized bottlebrush PVAs and observed that the increased molecular weight did not correspond to an increase in activity, unlike with linear polymers, and hence determined that steric confinement limits activity (Fig. 4d)^67^.

While PVA has been the most studied polymeric-IRI, the findings from small molecule IRIs suggest that hydroxyl groups are not essential components and that a wide chemical space should be studied. Also, PVA itself is a relatively challenging polymer to access, as vinyl acetate is not easy to polymerize by controlled RAFT methods, only a limited range of additional functionality can be included by co-polymerization and the removal of acetate groups requires harsh conditions. Inspired by this, Matsumura et al., reported that poly(ampholytes), polymers with mixed cationic and anionic groups, were remarkable cryopreservatives and seemed to influence ice crystal morphologies at high concentrations (Table 2)

**Table 2:** Polyampholytes’ ice recrystallization inhibition activity

	

*MGS* mean grain size determined as the average area of each ice crystal; *MLGS* mean largest grain size, which reflects the length of the largest ice crystal

^68^. Mitchell et al. synthesized well-defined poly(ampholytes) via post-polymerization modification of poly(aminoethyl methacrylate) from RAFT polymerization. This revealed that when an exact 50:50 ratio of carboxylic acids to amines were present, the polymer showed clear IRI but excess of either charge removed activity^69^. PVA_10_, a relatively inactive chain length, is still around 6× more active than the most active ampholyte (10% MLGS vs. ~70% for ampholytes at the same concentration). This was, however, still a remarkable observation, in that there were no obvious ice-binding sites, and that charge balance appears to be crucial. A further feature is that the basic requirement of an ampholyte is very synthetically accessible; an equal balance of cations and anions. Matsumura and co-workers have reported copolymers of 2-(dimethylamino)ethyl methacrylate and methacrylic acid^70^ and also carboxylated polysaccharides (dextran) as IRI-active polymers^71^. To date, there is no clear mechanistic explanation for why these work. Due to the synthetic routes used, only on average, were these materials actually 1:1 cationic to anionic, and the sequence distribution within the polymers was random. To overcome both these challenges, maleic anhydride copolymers have been exploited; maleic anhydride has a low propensity to self-propagate, and hence when a second monomer is added, near-perfect alternative (ABABx) structures are formed. Furthermore, the anhydride ring can be opened with a nucleophile to install the desired functionality. Mitchell et al^72^. and Stubbs et al. have exploited this to generate libraries of sequentially modified poly(ampholytes) to probe their function^73^. Increasing the backbone hydrophobicity lead to increased IRI activity, but at a cost of significantly reduced solubility—the role of hydrophobicity is again crucial to highlight here. It was found that dimethyl amino side chains lead to the most active polymers, despite being less hydrophobic than ethyl and propyl analogues; this subtle balance between hydrophobicity and hydrophilicity underlies the challenges in the rational design of AFP mimics. These polymers, while more active than the ampholytes previously mentioned, are still much less active than PVA of a similar molecular weight. Interestingly, these sequence-regulated polymers appeared to have higher activity than random copolymers but again the fundamental mechanisms governing activity remains unknown^73^. Other synthetic materials have also been investigated as cryoprotectants, with suggested IRI activity, but are only just emerging. Graphene oxide has also been shown to have IRI activity, with its ability to inhibit ice growth demonstrated as being as effective as PVA at similar concentrations^74^.

## Ice Nucleating Proteins and Materials

**Fig. 5:**
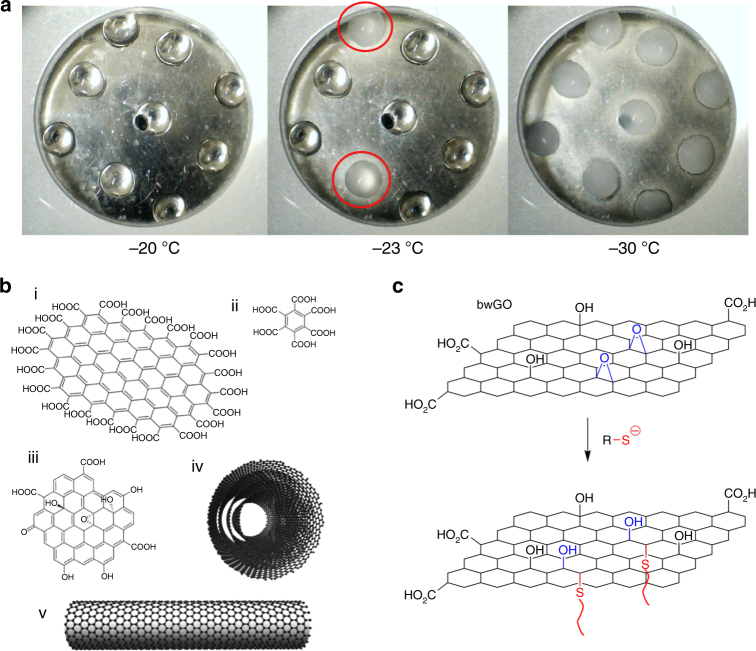
Ice nucleating materials. **a** Multi-point freezing assay used to assess ice nucleation, nucleating droplets are circled in red^82^; **b** Carbon nanomaterials with ice nucleation activity: (i) carboxylated graphene nanoflakes, (ii) mellitic acid, (iii) graphene oxide, (iv) multiwalled carbon nanotubes and (v) single walled carbon nanotubes^81^; **c** Graphene oxide-polymer composites^82^. **a** and **c** are reproduced from Biggs et al. (2017)^82^ with permission of PCCP owner societies. These images are not included under the creative commons licence for this article. **b** is reproduced from Whale et al. (2015)^81^ published under CC-BY4.0 at ACS

In addition to the AF(G)Ps, which inhibit the growth of ice crystals, there are also ice nucleating (formation) proteins. It is important to include a brief discussion of these, as the promotion of nucleation by biological macromolecules is also an interesting, and still a misunderstood process with many potential applications and scope for synthetic materials chemists to mimic. The most widely used ice nucleating protein is from *Pseudomonas syringae* which promotes frost formation on plant leaves, to release nutrients for feeding^76^. As with AF(G)Ps the mechanism of action is also not clear but water ordering is thought to be involved^77^. The underlying mechanism of ice nucleation (even in the absence of promoters) is still not understood and is a major challenge in modelling and theory^78^, made harder by the lack of sequentially modifiable materials which can be used to carry out systematic structure-activity studies and the fact that, due to the stochastic nature of ice nucleation, time-consuming multi-point assays must be used (Fig. 5a). Currently a number of materials, including soots^79^, have been shown to promote ice nucleation. Whale et al. have reported the first synthetic materials which can promote nucleation; carbon nanotubes, graphene nanoflakes and associated structures (Fig. 5b)^80^. Covalent modification of the surface of base-washed graphene oxide enabled the nucleation temperature to be tuned over a 15 °C range (Fig. 5c)^81^. The exact link between ice nucleators and antifreezes remains unclear, but it has been shown that synthetic AFGP mimics also demonstrate some nucleation inhibition properties^82^. In short, this process remains open for study and is even less studied than the already complex IRI mimetics.

## Enhanced Cell Cryopreservation with Ice Recrystallization Inhibitors

Tissue engineering, gene therapy, and cell transfusion all rely on the ability to store and transport cells and tissues in order to be clinically successful^83^. Cryopreservation is the most successful method for storage of these biological materials but is a complex process in which the sample volume, cooling rates, and cryoprotectants, such as dimethyl sulfoxide (DMSO), are all extremely important. Unfortunately, DMSO exhibits cytotoxicity at room temperature^84, 85^, damages mitochondrial integrity^86^, has been shown to impact the epigenetic profile of cells^15, 87^, disrupts intracellular cell signalling^88^, and if transfused into patients leads to a vast range of clinically undesirable symptoms. Due to this, there is a real need for new cryoprotectants with innovative modes of action, and AF(G)P inspired materials are presenting an exciting new avenue.

The formation of ice under typical cryopreservation conditions is inevitable and in most cases ice will form outside the cell^83^. Formation of extracellular ice creates an increased osmotic pressure across the cell membrane and this “osmotic flux” intensifies as ice growth continues after the nucleation event; cells will rupture if they cannot dehydrate fast enough. However, it has been shown that dehydration along with exposure to severely high concentrations of solutes is also lethal to the cell and will facilitate irreparable damage to the cell membrane^89^. Penetrating cryoprotectants, such as DMSO, readily cross the cell membrane and decrease the concentration of intracellular electrolytes while maintaining greater cell volumes but still carry with them the cytotoxic effects mentioned previously. Non-penetrating cryoprotectants do not cross the cell membrane, which results in an increase of osmolarity in the extracellular solution, facilitates dehydration prior to freezing, and prevents the formation of intracellular ice. Therefore, controlling and limiting extracellular ice growth, should be able to modulate and improve cell survival.

In 1992, Carpenter and Hansen demonstrated that addition of AFPs to blood cryopreservation solutions increased the post-thaw recovery levels. However, the effect was limited, as the AFP concentration was increased, the cell recovery actually decreased^90^. This was found to be due to the secondary effect of dynamic ice shaping, promoting bipyramidal ice crystals which could pierce the cell membranes. This was further demonstrated by Capicciotti et al. who showed various AF(G)Ps in the presence of 5% DMSO resulted in good cell post-freeze viabilities but cells frozen with a LpAFP mutant, which had a much smaller thermal hysteresis gap (and hence less dynamic ice shaping activity), resulted in the least amount of cellular damage^91^. In addition to ice-shaping, a challenge of using native AF(G)Ps is the potential for immunogenicty and toxicity. AFGPs have been found to have some cytotoxicity against human cell lines^16^ and conflicting reports showing either benefits or problems^12, 92, 93^ of AFGPs in cryopreservation, and hence polymer mimics with specific IRI (and not TH/DIS) could make a major impact.

**Fig. 6:**
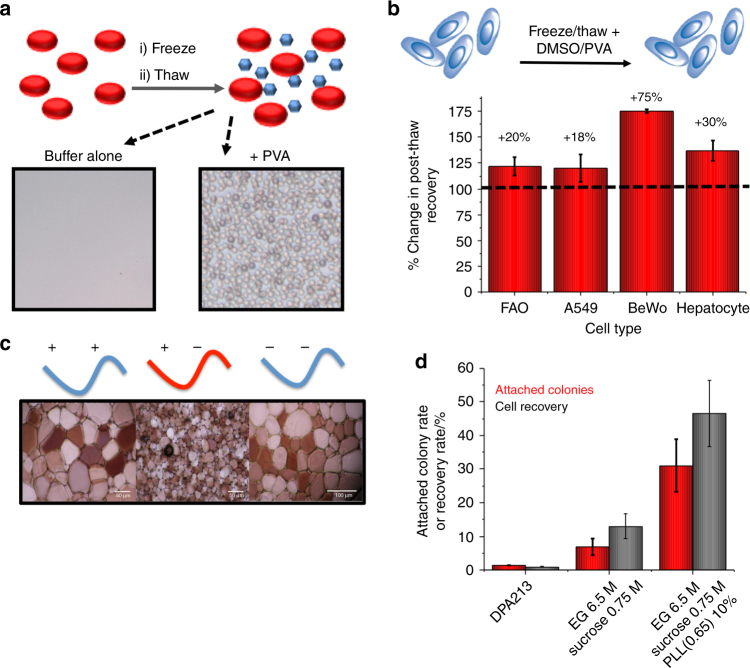
Cryopreservation of cells using IRI-active polymers. **a** Solvent-free red blood cell cryopreservation upon addition of 1 mg mL^−1^ PVA. Micrographs show recovered intact red blood cells; **b** Somatic cell recovery post-thaw in PVA/DMSO mixtures; **c** Polyampholyte IRI activity as a function of charge balance; **d** Cryopreservation of stem cells using ethylene glycol (EG) supplemented with polyampholytes and sucrose^102^. **a** is adapted from Deller et al. (2014)^14^. **b** is adapted from Deller et al. (2016)^97^ published under CC-BY3.0 at RSC. **c** is adapted from Mitchell et al. (2014)^70^ with permission from the Royal Society of Chemistry. This image is not included under the creative commons licence for this article. **d** is adapted from Matsumura et al. (2011)^102^. Copyright (2011) Elsevier Inc. This image is not included under the creative commons licence for this article

Gibson et al., showed that addition of PVA to red blood cells cryopreservation mixtures significantly enhances cryopreservation outcomes, either alone, or in tandem with hydroxyl ethyl starch or polymeric hydrogelators (Fig. 6a)^14, 94, 95^. PVA also improved the protection of A549 and BeWo cells during DMSO-mediated cryopreservation, with PVA alone not enabling recovery of nucleated cells (Fig. 6b)^96^. It is important to note that PVA also has an effect of inhibiting nucleation, and can improve cryopreservation by vitrification by reducing de-vitrification^97^. However, as vitrification requires very high levels of solvents (often >40 wt%) this method is not as widely used for cells, with the slow-thawing method being preferred, but has been used to cryopreserve a rabbit kidney^98^. In addition to PVA, polyampholytes have been found to be extremely potent cryopreservation enhancers, despite their significantly lower IRI activity, relative to PVA or AF(G)Ps^69^. L929 and RMSCs cells were cryopreserved using polyampholytes as the sole CPA, which appear to directly interact with cell membranes, in addition to slowing ice growth^68^ which is similar to the proposed mechanisms of AF(G)P^33, 99, 100^. Ampholyte cryopreservation is optimal with an exact balance of cationic to anionic groups^101^, which also maps to IRI activity (Fig. 6c), which raises the question of just how much IRI is needed to enhance cryopreservation, or what other structural features are essential. Leclère et al. determined *C*-linked analogues of AFGPs protected WRL 68 cells during cryopreservation as efficiently as 2.5% DMSO^102^. Briard et al. found that several low molecular weight carbohydrate derivatives were effective IRIs and effective additives for cryopreservation of human red blood cells resulting in significantly higher numbers of intact red blood cells post-thaw, while using reduced quantities of glycerol^103^.

The above observations demonstrate that AF(G)P mimetics can clearly improve cryopreservation outcomes to increase cell yield post-thawing. However, for most cases, there is still the need for low molecular weight cryoprotectants/osmolytes to ensure efficient cryopreservation. Yet another challenge in this field is the question of how much IRI (or TH) activity is needed to enhance cryopreservation and how little cryoprotectant can be used while still achieving a clinically/biotechnologically useful recovery rate. If these questions can be answered, AF(G)P mimetics could have a huge impact, especially in emerging regenerative applications and the storage of primary stem cells, or those emerging from stem-cell factories.

## Summary

This focused review has summarized the current state-of-the-art in the rapidly emerging field of synthetic macromolecules which can mimic the function of antifreeze (glyco) proteins. In particular, recent developments in polymer chemistry have enabled a wide range of polymers to be accessed and although identification of any new material which is active remains rare, an increasingly large number are being discovered. It has become clear that the precise primary sequence and 3-D structure of natural antifreezes is essential for thermal hysteresis and ice shaping to occur, supporting a molecular-recognition type mechanism on the ice crystal surface. However, ice recrystallization inhibition appears to be an easier to access property, with a diverse range of polymers, supramolecular assemblies and even small molecules being reported, despite no structural similarities between these. The application of IRIs in cellular cryopreservation is highlighted, where slowing the growth of ice during thawing has been found to lead to remarkable enhancements in cell recovery post-storage proving innovative solutions to the logistical challenges associated with emerging regenerative medicine treatments. These advances have triggered renewed interest in this area, and we anticipate that as more research groups enter this exciting, highly interdisciplinary, field advances will continue to the point where synthetic materials may be able to outperform natural proteins, but with both increased selectivity (in terms of antifreeze properties) and also additional, currently unforeseen, functionality. We would suggest that (non-exhaustively) the following key challenges need to be addressed for the field to continue to advance:

- Do several molecular-level mechanisms give rise to the observed macroscopic effects and if so can these be separated?
- Do polymeric mimics function by the same mechanisms as AF(G)Ps?
- What is the exact role of amphipathicity in IRI?
- Is there a minimum/maximum IRI activity required to increase cell cryopreservation?
- Can AF(G)P mimics be used to remove the need for organic solvents in cryopreservation?
